# Impaired hypoglycaemia awareness in type 1 diabetes: lessons from the lab

**DOI:** 10.1007/s00125-018-4548-8

**Published:** 2018-02-07

**Authors:** Alison D. McNeilly, Rory J. McCrimmon

**Affiliations:** Division of Molecular and Clinical Medicine, Mailbox 12, Level 5, Ninewells Hospital and Medical School, University of Dundee, Dundee, DD1 9SY UK

**Keywords:** Counterregulatory responses, Habituation, Hypoglycaemia, Impaired awareness of hypoglycaemia, Mechanisms, Review, Type 1 diabetes

## Abstract

Hypoglycaemia remains the most common metabolic adverse effect of insulin and sulfonylurea therapy in diabetes. Repeated exposure to hypoglycaemia leads to a change in the symptom complex that characterises hypoglycaemia, culminating in a clinical phenomenon referred to as impaired awareness of hypoglycaemia (IAH). IAH effects approximately 20–25% of people with type 1 diabetes and increases the risk of severe hypoglycaemia. This review focuses on the mechanisms that are responsible for the much higher frequency of hypoglycaemia in people with diabetes compared with those without, and subsequently how repeated exposure to hypoglycaemia leads to the development of IAH. The mechanisms that result in IAH development are incompletely understood and likely to reflect changes in multiple aspects of the counterregulatory response to hypoglycaemia, from adaptations within glucose and non-glucose-sensing cells to changes in the integrative networks that govern glucose homeostasis. Finally, we propose that the general process that incorporates many of these changes and results in IAH following recurrent hypoglycaemia is a form of adaptive memory called ‘habituation’.

**Electronic supplementary material:**

The online version of this article (10.1007/s00125-018-4548-8) contains a slideset of the figures for download, which is available to authorised users.

## The clinical importance of impaired hypoglycaemia awareness

The characteristic symptom complex that alerts an individual to hypoglycaemia is well described and is broadly represented by three distinct symptom clusters, namely ‘autonomic’ (e.g. sweating, palpitations), ‘neuroglycopaenic’ (e.g. confusion, drowsiness) or ‘general malaise’ (e.g. headache, nausea) [1]. It is also widely reported that this hypoglycaemia symptom complex varies considerably both between individuals and for any given individual over the time course of their disease. For some individuals, the symptomatic response to hypoglycaemia changes markedly such that hypoglycaemia symptoms are not triggered until glucose levels are very low and often occurring after cognitive function is impaired. This is referred to as impaired awareness of hypoglycaemia (IAH), defined as ‘a diminished ability to perceive the onset of acute hypoglycaemia’ [2]. It is not a condition that is either present or absent in an individual but reflects a continuum in which differing degrees of IAH can occur and can vary over time. IAH affects 20–25% of all people with type 1 diabetes, and as much as 50% of those who have experienced severe hypoglycaemia. Worryingly, the incidence of IAH has not changed in the last two to three decades, even with the introduction of insulin analogues and improved insulin delivery systems [3]. The important clinical consequence of this is that, in type 1 diabetes, IAH results in a six- to eightfold increased risk for severe hypoglycaemia (defined as the need for external assistance to recover) [3, 4], which has a well-recognised impact on morbidity and mortality in those with type 1 diabetes [5] and places a significant psychosocial burden on family members involved with their care [6]. IAH also occurs in type 2 diabetes, affecting up to 10% of patients with insulin-treated type 2 diabetes and markedly increasing risk of severe hypoglycaemia [7].

In this review and the accompanying reviews by Iqbal and Heller [8] and Choudhary and Amiel [9], we outline our current understanding of why people with type 1 and long-duration type 2 diabetes develop IAH and consider the options that are currently available to prevent IAH or restore hypoglycaemia awareness. Specifically, in this review, we briefly outline the principal reasons why people with diabetes are prone to hypoglycaemia and discuss the mechanisms that may contribute to the development of IAH. We also propose the hypothesis that IAH may result from a special form of adaptive memory called ‘habituation’.

## Why do people with diabetes develop hypoglycaemia?

Although hypoglycaemia can occur in people without diabetes (e.g. ‘reactive hypoglycaemia’), it is not common and, with the exception of hypoglycaemia occurring during severe sepsis or malnutrition, it is not usually severe or of potential pathological consequence. As a fuel, glucose is so fundamental to the survival of an organism that multiple systems are in play to ensure that a continuous supply of glucose is provided to the tissues of the body. These systems act in concert to ensure that glucose utilisation by the brain, liver, muscle and adipose tissue (white and brown), and glucose production and release into the blood stream by the liver and kidney, are tightly regulated. It seems highly likely that these systems do not exist in isolation but form parts of a highly integrated network designed both to monitor overall blood nutrient levels as well as fuel stores, and to direct fuels where and when needed to specific tissues. For instance, a liver–brain axis that is responsive to liver glycogen content was recently demonstrated to modulate the counterregulatory response (CRR) to insulin-induced hypoglycaemia [10]. Readers are referred to an excellent review by Watts and Donovan, which elegantly describes how the glucose-sensing network is structured very much like a classical sensory–motor neural circuit with glucose as an internally sensed stimulus and the CRR as the motor output [11]. Brainstem and forebrain integrative centres then serve to coordinate signals for multiple peripheral and central outputs to ensure that glucose homeostasis is maintained [11].

In type 1 diabetes (and, to some degree, type 2 diabetes), hypoglycaemia is, by contrast, relatively common. There are three principal abnormalities that underlie this propensity to hypoglycaemia: (1) failure to clear circulating insulin during hypoglycaemia; (2) loss of normal pancreatic alpha cell responses; and (3) lower glucose threshold for release of counterregulatory hormones. Generally, the first two defects explain why low glucose levels develop more frequently in people with type 1 and type 2 diabetes in comparison with people without diabetes, while the third major defect (discussed in more detail in the next section) explains why a subset of people with type 1 and type 2 diabetes may be even more prone to hypoglycaemia and will occasionally suffer severe hypoglycaemia.

The CRR is initiated when glucose levels fall beneath the normal range and is designed to restore normal glucose homeostasis. It encompasses hormonal, symptomatic and behavioural responses. In people without diabetes, the first response to a decline in blood glucose is a reduction in insulin secretion that begins while plasma glucose concentration is still in the physiological range (~4.4 mmol/l) [12]. In contrast, insulin release from an unregulated subcutaneous depot in type 1 diabetes and/or the continued action of sulfonylureas in a non-glucose dependent manner in type 2 diabetes means that systemic insulin levels remain relatively high during hypoglycaemia in diabetes. This is the first of the CRR defects, namely the failure to clear (dissipate) insulin from systemic circulation during hypoglycaemia. Relative insulin excess increases glucose uptake and suppresses hepatic glucose production despite development of hypoglycaemia, and also acts peripherally to limit lipolysis and the delivery of gluconeogenic substrates to the liver. The overall effect is to increase glucose uptake by tissues and reduce its production, compounding any glucose-lowering stimulus.

**Fig. 1:**
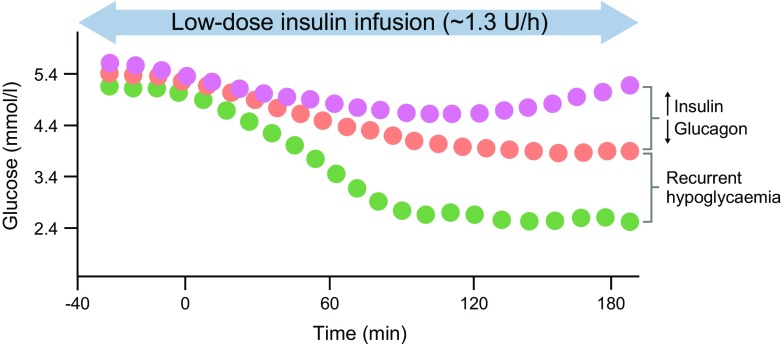
Counterregulatory failure in type 1 diabetes. Glucose profiles that might develop following exposure to a constant low-dose insulin infusion (1.3 U/h) in people with and without type 1 diabetes. Insulin infusion in people without diabetes (purple circles) leads to an initial fall in glucose before a CRR is triggered, resulting in glucose recovery towards baseline levels. In comparison, in people with conventionally treated type 1 diabetes (orange circles), glucose will fall further when exposed to the same insulin infusion and recovery will be impaired, mainly owing to a combination of relative hyperinsulinaemia and loss of a significant glucagon CRR. In contrast, following intensive insulin therapy (green circles) and exposure to recurrent hypoglycaemia, glucose levels are expected to fall much further and glucose recovery is markedly impaired. Figure generated based on data in [23]. This figure is available as part of a downloadable slideset

The second major CRR defect is loss of physiological glucagon secretion, which is profound in type 1 diabetes [13] but also present in longer-duration type 2 diabetes [14]. The pancreatic beta and alpha cell react in a synchronous way to changes in blood glucose levels (to which they are exposed) and the resulting insulin:glucagon ratio in the portal venous system is the major determinant, under most circumstances, of hepatic glucose production. In people without diabetes, developing hypoglycaemia leads to the suppression of endogenous insulin secretion (insulin ‘switch-off’) and a parallel rise in glucagon release from the pancreatic alpha cell. Paradoxically, by year 5 of disease duration in type 1 diabetes [13, 15] and in long-duration, reduced C-peptide type 2 diabetes [14], glucagon secretion during hypoglycaemia is markedly impaired. There is also a small, paradoxical increase in glucagon release in the postprandial state [16]. The reason why physiological alpha cell glucagon secretion is reversed in diabetes is currently unknown. The earlier and more profound effect in type 1 diabetes and the association with C-peptide levels suggest loss of regulatory beta cell signals, such as zinc, insulin or γ-aminobutyric acid (GABA) [17], but direct effects of glucose [17] or paracrine signals, such as basal hypersecretion of somatostatin, may also explain the loss of physiological glucagon [18]. Intriguingly, there has been recent interest in the role of the pancreatic delta cell, and in particular its major exocrine product, somatostatin, in this loss of normal glucagon regulation and this has led to the proposal that type 1 diabetes should perhaps be considered a disease of beta, alpha and delta cells [19]. In addition, the difference in glucose thresholds for insulin suppression (~4.4 mmol/l) and glucagon activation (~3.8 mmol/l) suggest a potential role for a central neurally mediated signal [17, 20, 21] in contributing to this defect. This reversal of normal pancreatic beta and alpha cell physiological responses to a reduction in glucose is the second major underlying reason for people with diabetes being so much more prone to more profound hypoglycaemia than those without diabetes (Fig. 1).

The third major CRR defect in type 1 and 2 diabetes is observed in individuals with IAH. Characteristic features include a higher threshold (lower glucose) for release of counterregulatory hormone and symptom responses to experimentally-induced hypoglycaemia, as well as reduced magnitude of these responses [22]. This is illustrated in Fig. 1, which shows that, when subjected to an identical glucose-lowering stimulus (a constant low-dose insulin infusion), participants with type 1 diabetes who had undergone 2–6 months of intensive insulin therapy [resulting in a change in HbA_1c_ from 81 ± 12 mmol/mol (9.6 ± 1.1%) to 54 ± 8 mmol/mol (7.1 ± −0.7%] demonstrated markedly suppressed counterregulatory hormonal and symptomatic responses to hypoglycaemia compared with baseline. This resulted in far more profound and prolonged hypoglycaemia [23]. Importantly, for individuals with suppressed CRRs, the glucose level at which symptomatic awareness of hypoglycaemia occurs is very low (usually <3.0 mmol/l) and at a point where cognitive dysfunction can be detected (generally <2.8 mmol/l). The principal symptoms of hypoglycaemia also change and are predominantly neuroglycopaenic in nature. This reduced ability to detect hypoglycaemia is referred to as IAH and, when more profound, as hypoglycaemia unawareness. The main cause of this third counterregulatory defect is considered to be prior exposure to hypoglycaemia itself. Long duration of diabetes is also a risk factor for IAH, but it is not clear whether this is a result of recurrent, often silent hypoglycaemia, chronic hyperglycaemia or microvascular disease directly affecting glucose-sensing elements. Because most of the research has examined the effect of recurrent hypoglycaemia, this review will focus on this area.

## Why do people with diabetes develop IAH?

A series of human clamp studies in the 1990s demonstrated that prior hypoglycaemia in individuals with [24] or without type 1 diabetes [25] is sufficient to significantly impair CRR to hypoglycaemia that was induced the following day. Similar responses to recurrent hypoglycaemia have been demonstrated in the rat [26] and mouse [27], indicating a highly conserved response to repeated glucose deprivation. In summary, repeated hypoglycaemia in diabetes leads to a progressive reduction in the CRR, which eventually results in IAH and a paradoxical increased risk of severe, disabling hypoglycaemia. We will now consider the mechanisms that may underpin this third CRR defect.

**Fig. 2:**
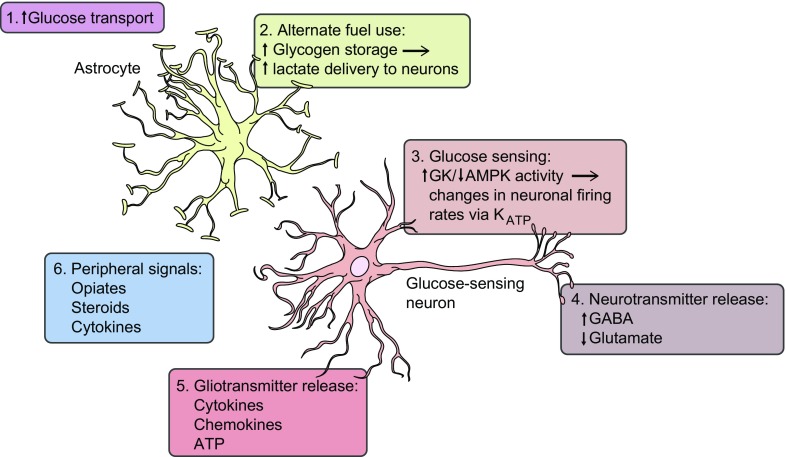
Potential mechanisms underlying the development of IAH. Multiple mechanisms may contribute to the development of IAH. The figure shows the relationship and communication between astrocytes and neurons to illustrate this, but peripheral glucose-sensing systems and peripheral organs may also contribute to the development of IAH. Proposed mechanisms include: (1) an increase in glucose transport through glucose transporters in the blood–brain barrier and/or on neurons/astrocytes; (2) increased glycogen storage within astrocytes (‘glycogen supercompensation’), leading to increased delivery of lactate to neurons; (3) increased activity of glucokinase (GK) or reduced activity of AMPK, leading to changes in neuronal firing rates, mediated by sulfonylurea receptor-1 (SUR-1)-selective K_ATP_ channels; (4) changes in neurotransmitter release, resulting in increased GABAergic tone (increased inhibitory GABA release and/or reduced excitatory glutamate release); (5) release of gliotransmitters, such as cytokines and chemokines, that modulate neuronal firing rates; and (6) peripherally (or centrally) generated signals, such as opiates/steroids and cytokines, that modulate neuronal firing rates. This figure is available as part of a downloadable slideset

While recurrent hypoglycaemia is recognised as the primary risk factor leading to the development of IAH, the underlying mechanism(s) responsible for this remain less well understood. There is an extensive literature that has examined this question and Fig. 2 illustrates potential mechanisms that may contribute to the development of IAH. It is important to note that this figure shows the relationship and communication between astrocytes and neurons to illustrate these concepts, but that this by no means excludes contributory roles for peripheral glucose-sensing systems, such as the portomesenteric vein sensors, or for peripheral organs, such as the adrenal gland, in the development of IAH. Hypoglycaemia represents profound cellular stress and, so, it is highly likely that multiple cell types will respond to this challenge at some level. As noted in Fig. 2, potential overall mechanisms that may underpin the development of IAH include changes in glucose and/or alternate fuel transport, or fuel storage, changes in the intracellular metabolism of glucose and transduction of the energy signal into altered neuronal firing rates, changes in intracellular communication via autocrine, paracrine or endocrine signals or changes in neurotransmitter release. There is limited scope for discussing these in detail but the following section will outline some of the work that has been carried out in these areas and refer the reader to recent, more specific review articles.

The initial focus of investigators was to determine if recurrent hypoglycaemia resulted in an increase in glucose and/or alternate fuel transport, or fuel storage. Certainly, studies in rodent models and humans show an increase in the capacity to transport glucose or alternate fuels through the blood–brain barrier and into neurons and astrocytes [5, 28], as well as an increase in whole brain glucose uptake after prolonged hypoglycaemia [29]. Similarly, it was previously suggested there may be an increased capacity to store glycogen in the brain, providing a more readily accessible fuel source during subsequent hypoglycaemia [5, 28]. However, many of these studies were conducted in people or rodents without type 1 diabetes. More recent work suggests no increase in cerebral glucose metabolism during hypoglycaemia in people with type 1 diabetes and IAH [30] and no increase in brain glycogen stores [31]. Moreover, compared with control animals, detailed tracer studies in rodents indicate that, after recurrent hypoglycaemia, neuronal glucose metabolism is actually decreased during subsequent hypoglycaemia [32]. In summary, these studies suggest that recurrent hypoglycaemia leads to an increased capacity of the human brain to use glucose and alternate fuels, but it is not clear exactly how or where that fuel is being used or whether this is ordinarily operative under conditions of hyperinsulinaemic hypoglycaemia (for detailed review, see [28]).

Alternatively, it is possible that prior hypoglycaemia increases the capacity of the cell (neuron) to metabolise glucose, generating more ATP for any given glucose level, which would directly modulate ATP-sensitive potassium (K_ATP_) channels and shift thresholds for glucose-sensing neuron activation. Certainly, recurrent hypoglycaemia increases the activity of neuronal glucokinase [33], the key first committed step in glucose metabolism in the cell, while pharmacological or genetic manipulation of neuronal glucokinase directly modulates the CRR [22]. Similarly, genetic manipulation of hypothalamic AMP-activated protein kinase (AMPK), an intracellular fuel gauge, also directly modulates CRRs [34]. However, as noted in the preceding paragraph, the evidence in support of an overall change in glucose metabolism is at best mixed and so, while targeting these critical glucose-sensing kinases may be a useful approach to pharmacologically amplifying the CRR, it remains uncertain whether they are actually responsible for the development of IAH [22]. An alternative approach is to directly target the downstream mediator of the intracellular metabolic signal, namely the K_ATP_ channel. Based on prior extensive animal research [22], this was recently shown to be an effective approach for amplifying the CRR to hypoglycaemia in individuals with long-standing type 1 diabetes and reduced hypoglycaemia awareness [35] .

Neurotransmitters released from glucose-sensing neurons within their neural circuit eventually activate the CRR. Within key brain glucose-sensing sites, such as the ventromedial hypothalamus (VMH), the excitatory neurotransmitter glutamate [21] and the inhibitory neurotransmitter GABA [36] are considered to be critical to the CRR. Recurrent hypoglycaemia leads to an increase in GABAergic inhibitory tone in the VMH [36], which likely suppresses the excitatory glutamatergic outputs that drive the CRR [21]. GABA_A_ receptor activation can also suppress CRRs to subsequent hypoglycaemia in humans [37]. This offers the therapeutic opportunity of targeting GABA to augment CRRs in IAH. However, the difficulty with progressing this latter work towards clinical trials of hypoglycaemia prevention is that GABAergic neurons are the major inhibitory neurons throughout the brain and so blockade of GABA receptors, unless of very specific isoforms, is liable to have significant adverse effects. It is also not clear from studies to date whether recurrent hypoglycaemia directly acts on neurotransmitter production and/or release or whether these changes are just secondary to a different underlying mechanism.

More recently, there has been significant interest in discovering how cytokine, purine, steroid, opiate, β_2_-adrenergic or serotonergic signalling may play roles in the development of IAH [22, 38–42]. These extracellular signals are able to modulate firing rates of glucose-sensing neurons. They may do so by actions on neighbouring astrocytes, intraneuronal metabolism, K_ATP_ channel activity, or through modulation of neurotransmitter release. They are secreted either in an autocrine fashion from neurons, released by neighbouring astrocytes or secreted from peripheral tissues, such as muscle. This knowledge has been derived from laboratory-based research, which has enabled proof-of-concept clinical trials. For instance, the inhaled highly-selective β_2_-adrenoreceptor agonist formoterol fumerate was shown to amplify the CRR to hypoglycaemia in a small group of participants with type 1 diabetes [42], while oral dehydroepiandrostenedione (DHEA), which has both anti-corticosteroid and anti-GABA activity, blunted the suppressive effect of recurrent hypoglycaemia on subsequent hypoglycaemia CRR in individuals without diabetes [39]. It is interesting that many of these extracellular signals help coordinate the body’s response to a wide variety of physiological stressors and play roles in both amplifying the acute response to a given stressor and initiating subsequent adaptive responses that are designed to protect cells during subsequent exposure to that stressor. Recent work from our own group, for instance, has shown that pro- and anti-inflammatory cytokine release during hypoglycaemia is modified significantly by prior exposure to hypoglycaemia and also type 1 diabetes [43]. This raises the possibility that the progressive suppression of CRR to hypoglycaemia may result, at least at a cellular level, from a series of adaptive responses to recurrent hypoglycaemia that is initiated by a cell to ensure that it is better able to withstand (tolerate) future exposure to a similar period of glucose deprivation. This concept is consistent with findings from animal models that show that prior hypoglycaemia actually limits the impact of subsequent very severe hypoglycaemia on neuronal survival [44], as discussed in more detail below.

## Habituation and impaired hypoglycaemia awareness

In a seminal review paper in the 1960s, Thompson and Spencer sought to describe the properties of a form of learning called ‘habituation’. Habituation is defined as a reduction in the psychological, behavioural or physiological response to a stimulus as a result of repeated or prolonged exposure. Habituation is a form of adaptive learning and is usually seen in the context of innate behaviours, presenting as a reduced response to repeated external or internal stimuli [45, 46].

Habituation is now recognised to occur in virtually all organisms in response to a variety of stressful stimuli [46]. At a biological level, habituation reflects a survival response where cells adapt to repeated exposure to the physiological stressor in order to develop greater tolerance of that specific stressor. There are clear parallels between habituation and IAH, with similar widespread effects of recurrent hypoglycaemia on physiological (e.g. suppressed counterregulatory hormonal and symptomatic responses), behavioural (e.g. reduced drive to feed) and psychological (e.g. reduced anxiety) responses. Thompson and Spencer [45] proposed nine principal features of a habituated response (updated more recently by Rankin et al. [46]). The first of these principles was: ‘Given that a particular stimulus elicits a response, repeated applications of the stimulus result in decreased response (habituation)…’. This is a characteristic feature of hypoglycaemia, where repeated exposure to hypoglycaemia leads to decreased symptoms and CRRs to a subsequent episode [24, 25]. Likewise, recovery of CRR through hypoglycaemia avoidance [47] is in keeping with the second characteristic of habituation; namely, ‘if the stimulus [hypoglycaemia] is withheld, the response [CRR] tends to recover over time (spontaneous recovery)’. In fact, studies in rodent and human models of IAH have reported findings consistent with six of the nine principles of habituation proposed by Thompson and Spencer (for review see [48]).

**Fig. 3:**
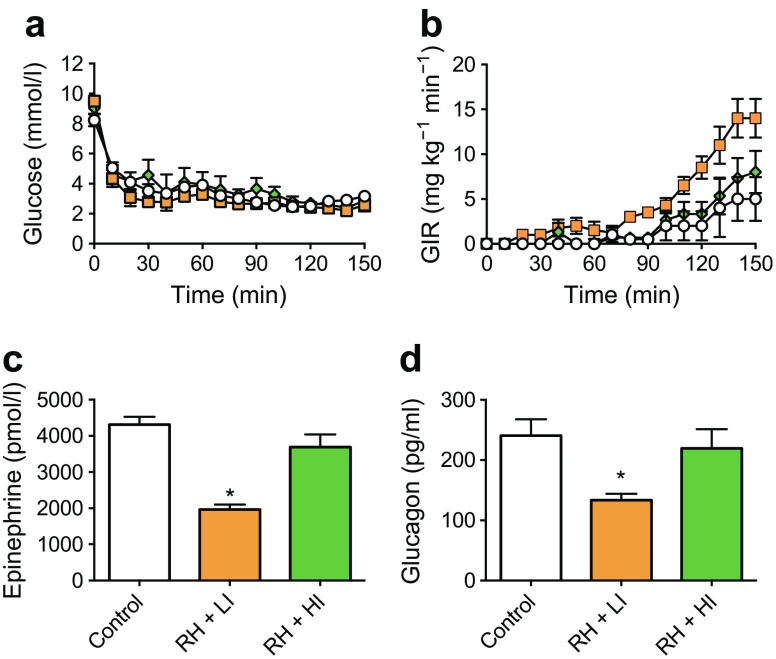
Dishabituation with high-intensity exercise. Rodents were subjected to recurrent episodes of insulin-induced hypoglycaemia or saline control injections over 4 weeks. Rats were then exposed to low-intensity or high-intensity exercise, the latter being a dishabituatory stimulus. Twenty-four hours later, animals underwent a hyperglycaemic clamp and CRR variables were measured. (**a**) Plasma glucose during hyperinsulinaemic–hypoglycaemic clamp study. (**b**) Glucose infusion rates (GIR) required to maintain equivalent hypoglycaemia. (**c**) Plasma adrenaline (epinephrine), and (**d**) glucagon responses during hypoglycaemia. Control animals, white circles/bars; recurrently hypoglycaemic (RH) animals exposed to low-intensity (LI) exercise, orange squares/bars; RH animals exposed to high-intensity (HI) exercise, green diamonds/bars. Values shown as mean ± SEM. *p < 0.05 vs control. Figure adapted from [49]. Material from this publication has been used with the permission of ADA. Copyright and all rights reserved. This figure is available as part of a downloadable slideset

A further defining feature of a habituated response is that it should be possible to acutely restore the response through introduction of a novel, usually strong stimulus. This is a process referred to as ‘dishabituation’. In a recent study from the authors’ laboratory, introduction of a dishabituatory stimulus was indeed shown to restore normal CRRs to hypoglycaemia in a rodent model [49]. In this study, rodents were subjected to recurrent episodes of insulin-induced hypoglycaemia or saline control injections over a 4 week period. Subsequently, the rats were exposed to either a low-intensity exercise regimen on a treadmill or a burst of high-intensity exercise (dishabituatory stimulus). The following day, all animals underwent an equivalent hypoglycaemic stimulus (hypoglycaemic clamp). As expected, CRRs were suppressed in rodents exposed to 4 weeks of recurrent hypoglycaemia; however, in the group of rodents who had experienced recurrent hypoglycaemia followed by a single episode of high-intensity exercise, the CRR was restored to control levels (Fig. 3) [49]. This finding very much suggests that IAH results from habituation and is a learned adaptive response to repeated exposure to the same stimulus. This concept remains hypothetical and requires further validation in both laboratory-based in vitro and animal studies, and human studies but does provide a basis for conceptualising the whole organism response to recurrent hypoglycaemia. It may seem paradoxical that the organism responds to repeated hypoglycaemia by initiating an adaptive response that renders it more susceptible to future hypoglycaemia, but we should recognise that iatrogenic hypoglycaemia as a result of exogenous insulin or sulfonylurea exposure, in the presence of an added alpha cell glucagon secretory defect, creates a physiological condition that we have not evolved to respond to, namely profound hyperinsulinaemic hypoglycaemia.
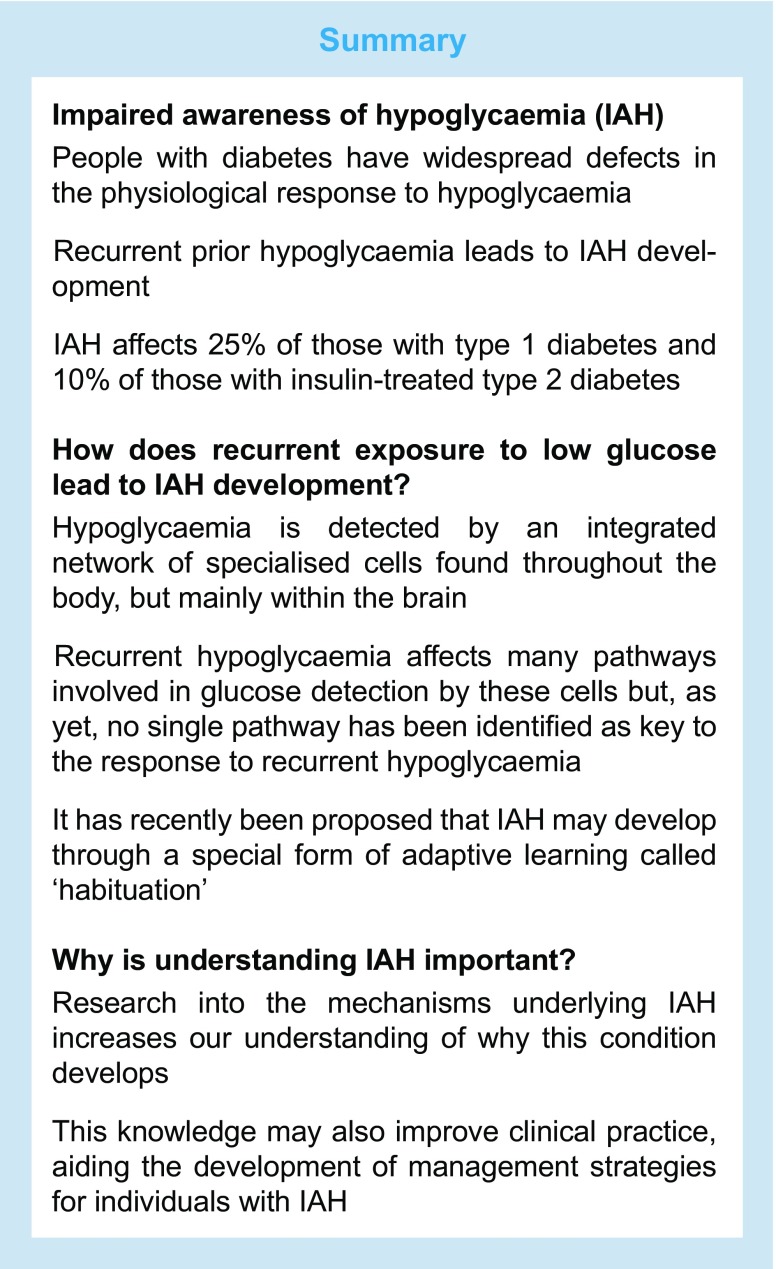

## Conclusions

Hypoglycaemia remains a concern for people with type 1 and type 2 diabetes. In diabetes, hypoglycaemia results from profound disturbances in glucose homeostasis and, in particular, the inability to ‘shut off’ exogenous insulin delivery and loss of normal physiological regulation of glucagon secretion. Recurrent hypoglycaemia leads to further marked suppression of counterregulatory hormonal and symptomatic responses to subsequent hypoglycaemia, a clinical phenomenon referred to as IAH. The mechanism(s) underlying the development of IAH are incompletely understood but may, or are perhaps likely, to involve multiple rather than single steps in the generation of a CRR. These adaptations occur on multiple levels, from within individual glucose and non-glucose-sensing cells, all the way up to the integrative networks that govern glucose homeostasis. Conceptually, IAH may result from habituation (a special form of adaptive memory) to recurrent hypoglycaemia. This form of adaptation to repeated hypoglycaemia is aimed at inducing greater cellular tolerance of subsequent hypoglycaemia, but in fact increases susceptibility to future hypoglycaemia.

By increasing our understanding of the mechanisms that lead to the development of IAH, we are better placed to consider how to approach the management of this condition. For instance, to resolve the problem of insulin dissipation, we are now aware that more physiological- and glucose-responsive insulin therapies are required. Restoration of physiological glucagon secretion would also be a major advance, but we must first obtain a clearer idea of why this defect develops in diabetes. Finally, habituation is best treated by avoidance of the habituating stimulus, namely hypoglycaemia, and this is currently best achieved through structured education, as discussed by Iqbal and Heller [8]. Further, for some individuals, habituation leads to chronic behavioural change that may require additional interventions, such as cognitive behavioural therapy, to support hypoglycaemia avoidance strategies, as discussed by Choudhary and Amiel [9]. In the future, novel strategies such as dishabituation or pharmacological interventions may also be an option as part of the package of care required to restore or reverse IAH.

## Electronic supplementary material

ESM (PPTX 486 kb)

## Abbreviations

CRR: Counterregulatory response
GABA: γ-Aminobutyric acid
IAH: Impaired awareness of hypoglycaemia
K_ATP_: ATP-sensitive potassium channel
VMH: Ventromedial hypothalamus
