# Supplementary material for: Impaired hypoglycaemia awareness in type 1 diabetes: lessons from the lab
Source: Diabetologia. 2018 Feb 7;61(4):743–50. doi: 10.1007/s00125-018-4548-8 (PMC6448989; doi:10.1007/s00125-018-4548-8)
Supplement: Supplementary file 1 — (PPTX 486 kb) [file 125_2018_4548_MOESM1_ESM.pptx]

## Slide 1
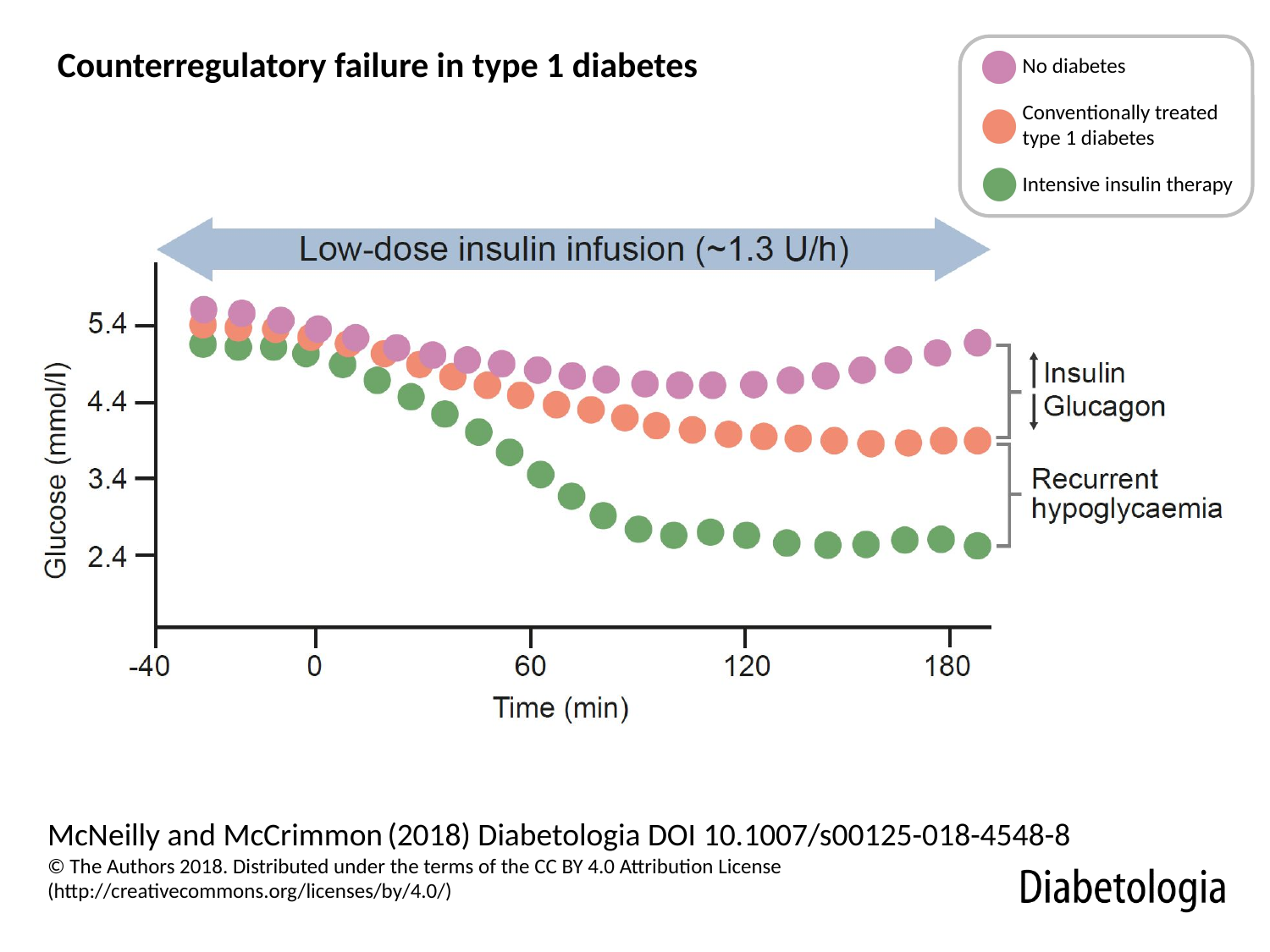

Counterregulatory failure in type 1 diabetes
No diabetes
Conventionally treated type 1 diabetes
Intensive insulin therapy
McNeilly and McCrimmon (2018) Diabetologia DOI 10.1007/s00125-018-4548-8
© The Authors 2018. Distributed under the terms of the CC BY 4.0 Attribution License (http://creativecommons.org/licenses/by/4.0/)

## Slide 2
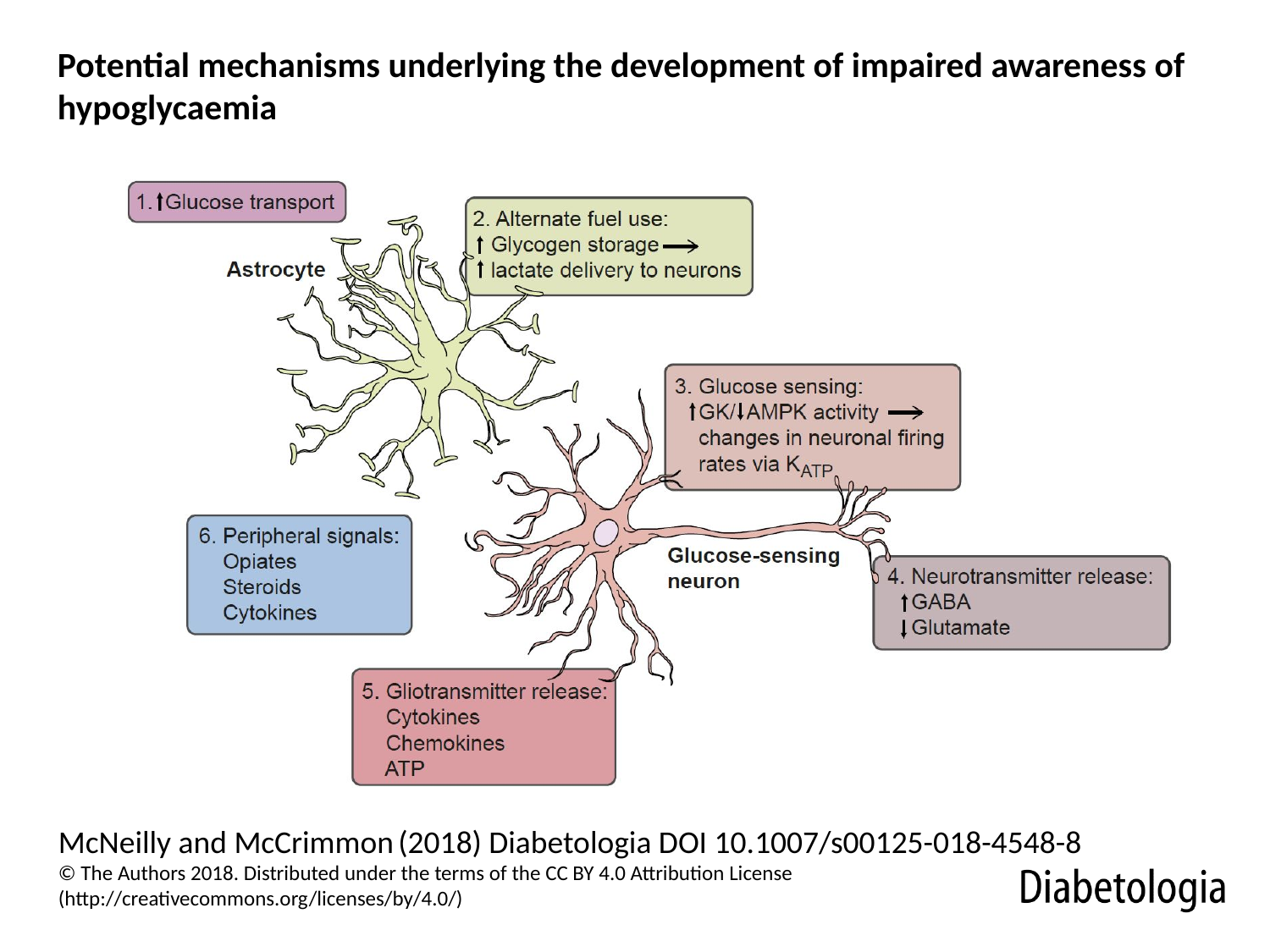

Potential mechanisms underlying the development of impaired awareness of hypoglycaemia
McNeilly and McCrimmon (2018) Diabetologia DOI 10.1007/s00125-018-4548-8
© The Authors 2018. Distributed under the terms of the CC BY 4.0 Attribution License (http://creativecommons.org/licenses/by/4.0/)

## Slide 3
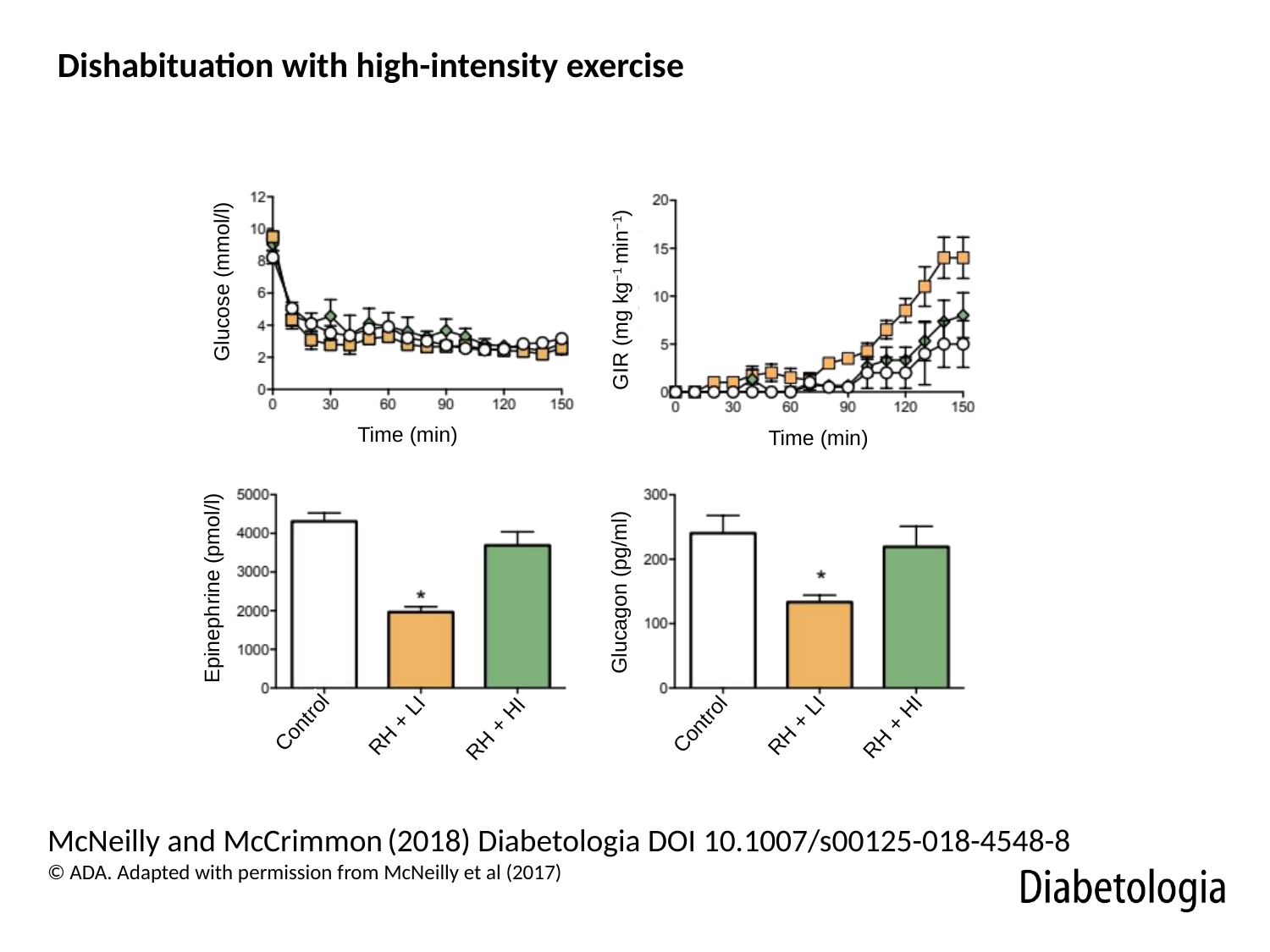

Glucose (mmol/l)
Time (min)
GIR (mg kg−1 min−1)
Glucose (mmol/l)
Time (min)
Time (min)
Epinephrine (pmol/l)
Glucagon (pg/ml)
Control
Control
RH + HI
RH + HI
RH + LI
RH + LI
Dishabituation with high-intensity exercise
McNeilly and McCrimmon (2018) Diabetologia DOI 10.1007/s00125-018-4548-8
© ADA. Adapted with permission from McNeilly et al (2017)
